# Fabrication of Novel Functional Cell‐Plastic Using Polyvinyl Alcohol: Effects of Cross‐Linking Structure and Mixing Ratio of Components on the Mechanical and Thermal Properties

**DOI:** 10.1002/gch2.202100026

**Published:** 2021-06-27

**Authors:** Kohei Iritani, Akihito Nakanishi, Ayami Ota, Takashi Yamashita

**Affiliations:** ^1^ Department of Applied Chemistry School of Engineering Tokyo University of Technology Tokyo 192‐0982 Japan; ^2^ School of Bioscience and Biotechnology Tokyo University of Technology Tokyo 192‐0982 Japan; ^3^ Graduate School of Bionics Tokyo University of Technology Tokyo 192‐0982 Japan

**Keywords:** biodegradable polymers, cell‐plastics, green algal cells, polyvinyl alcohol, water absorption ability

## Abstract

The current system of disposal of plastic materials fabricated from petroleum‐based resources causes serious environmental pollution. To solve the problem, a bioplastic called “cell‐plastic” is developed, in which unicellular green algal cells serve as a fundamental resource. This approach converts CO_2_ in the atmosphere directly into plastic products by exploiting the photosynthetic‐driven proliferation of algal cells. Herein, cell‐plastic films are fabricated using biodegradable and water‐soluble polyvinyl alcohol (PVA) as a matrix, in which the effects of a cell‐to‐matrix mixing ratio and the chemical structure of the matrix on the mechanical and thermal properties are investigated. As a method of the chemical structural change, a cross‐linking structure is introduced to the matrix by connecting hydroxy groups of PVA using aldehyde. The tensile tests reveal that the PVA‐cell‐plastic film maintains the mechanical properties of PVA film. Moreover, a cross‐linked cell‐plastic film exhibits high water absorption, making it suitable as a functional cell‐plastic material.

## Graphical abstract

Given the goal of reducing CO_2_ emissions and plastics pollution, a method to produce “cell‐plastics” is developed, whereby unicellular green algal cell serves as a raw material. In this research, polyvinyl alcohol (PVA) with biodegradability is used as a matrix to fabricate cell‐plastics with high mechanical strength. Moreover, cross‐linked PVA produces a functional cell‐plastic with high water‐retention.

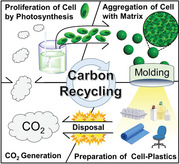

## 1 Introduction

**Figure 1:**
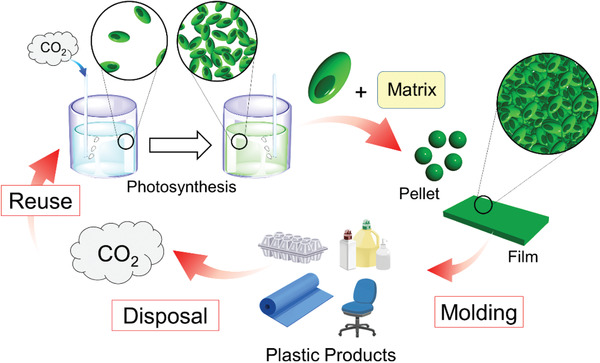
Schematic illustration of carbon circulation based on cell‐plastics.

Recent years has seen a growing worldwide interest in fabricating bioplastics such as biomass^[^ ^1^ ^]^ and biodegradable plastics,^[^ ^1^, ^2^ ^]^ which are biodegraded to H_2_O and CO_2_ under typical environmental conditions, to reduce the production of oil‐based plastics and replace them with bioplastics. Developing biodegradable plastics has been also attractive to reduce environmental pollution such as micro‐plastics, which critically damage ecosystems.^[^ ^3^ ^]^ Recently, blend materials that combine non‐biodegradable and biodegradable polymers have been studied to control the mechanical and physical properties of partially biodegradable materials.^[^ ^4^ ^]^ However, the numerous biodegradable plastics produced heretofore have been made from petroleum‐based polymers. In contrast, bioplastics made from biomass materials mixed with biodegradable polymers have also been developed.^[^ ^5^ ^]^ For example, Peng and co‐workers improved the mechanical strength of biodegradable polybutylene succinate (PBS) by mixing with a lignin derivative having a carboxy group as a donor or acceptor unit of hydrogen bonding with PBS.^[^ ^5e^ ^]^ Although these biodegradable plastic materials reduce the use of petroleum‐based components by using biomass resources, problems remain because of complications in plastic‐production stemming from the extraction, purification, and chemical treatment of resources of microbial origin.^[^ ^6^ ^]^ In one effort to advance the bioplastics, we fabricated “cell‐plastics” as novel bioplastics produced by unicellular green algal cells.^[^ ^1^, ^7^ ^]^ It would be expected that photosynthetic algae converted CO_2_ in the atmosphere directly into plastic materials without requiring any process preparing bioresources from algal cells.^[^ ^8^ ^]^ In the present work, we used a biodegradable polymer as a matrix to reinforce cell‐plastic materials because cell aggregation alone does not provide the requisite mechanical strength for daily use. Our ultimate goal is to fabricate biodegradable and carbon‐recycling plastics with the requisite mechanical and thermal properties for daily use (**Figure** **1**).

To improve the mechanical strength of aggregated cells, we previously produced cell‐plastics using a thin organic layer,^[^ ^7a^ ^]^ PBS,^[^ ^7b^ ^]^ and starch^[^ ^7c^ ^]^ as matrices. Although self‐standing films were obtained in each case, they lacked the mechanical strengths needed for daily use probably because of weak interactions between hydrophilic cells and hydrophobic polymers. Herein, we used polyvinyl alcohol (PVA)^[^ ^2^, ^9^ ^]^ for the cell‐plastic because PVA has water‐solubility exhibiting affinity with the hydrophilic cell as well as biodegradability. PVA is commonly used as a coating reagent, adhesive, or as a protective film against pesticides and detergents.^[^ ^10^ ^]^ Moreover, by adding a cross‐linking reagent such as aldehyde derivatives or inorganic salts, PVA transforms into a hydrogel in which water is captured in a 3D network structure formed by the cross‐linking between hydroxy groups on neighboring polymer chains.^[^ ^11^ ^]^ It is known that PVA hydrogels have potential for use in the horticultural and agricultural sector. In addition, bio‐PVA has also been produced from biomass resources,^[^ ^1a^ ^]^ so we expected that PVA may well serve as an appropriate matrix for cell‐plastics.

**Figure 2:**
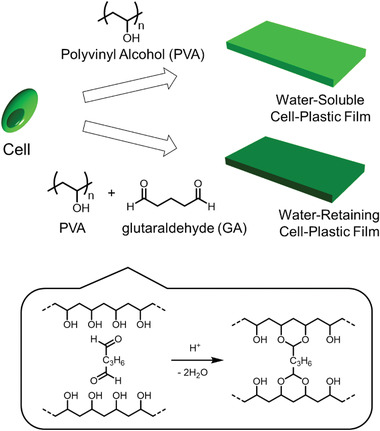
Scheme for fabrication of cell‐plastic films using PVA as the matrix.

To this end, we planned to fabricate PVA‐cell‐plastic films (**Figure** **2**) and investigate their mechanical and thermal properties to determine their suitability for daily use. In addition, to design cell‐plastics for a wide range of applications, we investigated the effects on the mechanical and thermal properties of the cell‐plastics affected by the mixing ratio of components and the formation of the cross‐linking structure to the matrix. As a cross‐linking reagent, glutaraldehyde^[^ ^11d^ ^]^ was used due to its relative environmental compatibility and the formation of covalent bonds leading to high thermal and chemical stability. As shown in Figure 2, the cross‐linking structure forms when two polymer chains are connected by two formyl groups attached to glutaraldehyde via acetalization by acid catalysis. Moreover, commercially available *Chlorella* sp. (called “cells” in this paper) served as unicellular green algal cells.

We previously reported that the mechanical strength of the cell‐plastics using a PBS matrix was found to be significantly less than that of a PBS film.^[^ ^7b^ ^]^ In contrast, in this work, we produced the PVA‐cell‐plastic film with a Young's modulus similar to that of the PVA film, which indicates that the affinity between cells and matrix affects the mechanical strength of the resulting cell‐plastic. In addition, it was revealed that the mechanical and thermal properties of the PVA‐cell‐plastic films were controlled by the cross‐linking structure in the matrix and varying the cell‐to‐PVA mixing ratio. Surprisingly, the cross‐linked PVA‐cell‐plastic film allows for significantly greater water content per initial mass of the film compared to that without cells, making this the first functional cell‐plastic material. These results open a route to fabricate sustainable and functional cell‐plastics for many useful purposes.

## 2 Results and Discussion

### 2.1 Preparations of PVA‐Cell Plastics

**Figure 3:**
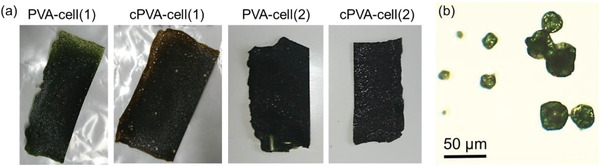
a) Photographs of PVA‐cell‐plastic films and b) optical microscopy image of *Chlorella* sp.

For the present research, we prepared four types of flexible and self‐standing PVA‐cell‐plastic films; films with a 1:1 (2:1) cell‐to‐PVA mixing weight ratio are called PVA‐cell(1) [PVA‐cell(2)], and their cross‐linked films are called cPVA‐cell(1) [cPVA‐cell(2)]. **Figure** **3** exhibits photographs of the films and an optical microscopy image of the cells, which shows that the cell agglomerations range in size from several micro meters to tens of micro meters. In addition, some spherical aggregations of cells reach the size of 100 µm (Figure S1, Supporting Information). Visual observation indicated that the cells were homogeneously dispersed throughout the film, with no PVA‐cell phase separation. To compare the mechanical and thermal properties with PVA‐cell films, PVA and cross‐linked PVA films were also manufactured (called “PVA” and “cPVA,” respectively; see Supporting Information).

**Figure 4:**
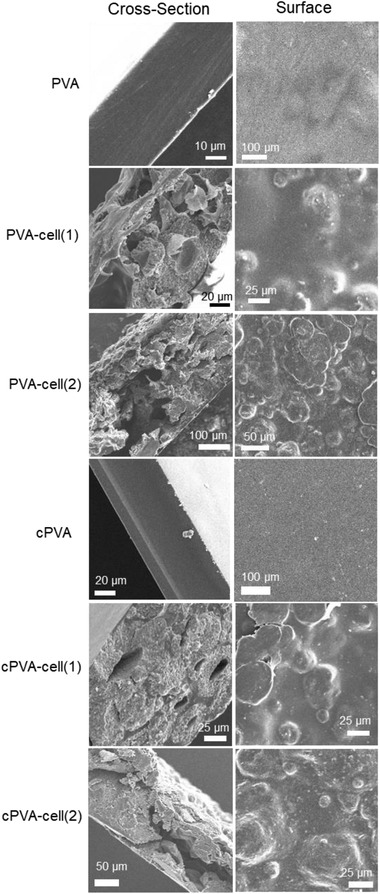
SEM images of cross‐section and surface of PVA and PVA‐cell‐plastic films.

Scanning electron microscopy (SEM) observations of the films were performed. **Figure** **4** shows SEM images of cross‐sections and surfaces of the films. These samples were broken naturally by pulling on the films at room temperature in the atmosphere. The cross‐section images indicated that PVA and cPVA films formed a high‐density material, whereas each PVA‐cell‐plastic film (with or without cross‐linking) formed a porous structure. Moreover, a fine split appeared in the cross‐sections of the PVA‐cell(1) and PVA‐cell(2) films, whereas the cPVA‐cell(1) and cPVA‐cell(2) films presented relatively planar faces with no fine split, which suggests that the cross‐linked films offer relatively high resistance to external stress compared with the non‐cross‐linked films. In addition, spherical cells appeared in the enlarged SEM cross‐section images of each PVA‐cell‐plastic film (Figure S2, Supporting Information), indicating that the cells maintain its particulate shape despite the addition of aldehyde and acid in the manufacturing process. On the other hand, the surface of the PVA‐cell‐plastic films were not planar but exhibited a random undulating structure probably due to the cell aggregations although homogeneous structures appeared on the surface of each PVA and cPVA film. These results leaded to the conclusion that the addition of cells modified the structure in the interior and on the surface of the final PVA‐cell‐plastic materials.

To evaluate the wettability of the surface, we measured the surface‐wetting contact angle of the cell‐plastic films. **Table** **1** summarizes the measured contact angles, and Figure S3, Supporting Information shows typical photographs of water deposited on the films. The cross‐linked PVA‐cell‐plastic films had greater contact angles than the corresponding non‐cross‐linked films, which is attributed to the reduction of the hydroxy groups in the polymer chains and increased hydrophobicity due to glutaraldehyde in the cross‐linked films. In addition, although the cell exhibits the high polarity, the PVA‐cell(1) and cPVA‐cell(1) films were more water repellent than the PVA and cPVA films, respectively, which suggested no decrease in the surface free energy of the PVA‐cell films probably because of their random undulating surface structure due to a distorted sphere of the cell. However, further addition of cells did not increase the contact angle. The tendency differs from our previous report on PBS‐cell‐plastics, for which the water repellency of the film increased according to the PBS‐cell ratio.^[^ ^7b^ ^]^ For this work, the precise contact angle is difficult to discuss because various effects contribute to the surface wettability such as hydrophilicity of the components, water repellency due to the cross‐linking structure, and the surface structure. However, despite these difficulties, the results clarify that the surface wettability of the PVA‐cell‐plastic film is determined by the chemical structure of the matrix and the PVA‐to‐cell‐plastic ratio.

**Table 1:** Contact angle of PVA and PVA‐cell‐plastic films

Film	Contact angle [°]
PVA	47.1 ± 6.7
PVA‐cell(1)	56.9 ± 4.1
PVA‐cell(2)	44.1 ± 3.5
cPVA	56.2 ± 8.9
cPVA‐cell(1)	75.0 ± 1.9
cPVA‐cell(2)	63.2 ± 4.0

### 2.2 Evaluations of Mechanical Properties of PVA‐Cell Plastics

**Figure 5:**
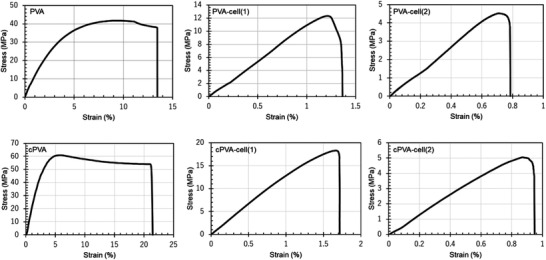
Typical stress‐strain curves for PVA‐cell‐plastic films.

Next, we evaluated the mechanical properties of each film based on tensile tests. **Figure** **5** shows typical stress‐strain curves for each film, and **Table** **2** lists the average Young's modulus and tensile strength. Note that, the PVA film has high mechanical strength due to low structural distortion by hydrogen bonding between the hydroxy groups in the polymer chains.^[^ ^11d^ ^]^ Surprisingly, Young's modulus of the PVA‐cell(1) film was competitive with that of the PVA film, although the Young's modulus decreased from 450 MPa for the PBS film to 240 MPa for the PBS‐cell‐plastic film (PBS : cell = 1:1) in the previous report.^[^ ^7b^ ^]^ This result was attributed to the different affinity between cells and matrix; in other words, hydrophilic PVA experiences high affinity with the cell‐plastic. Unfortunately, the tensile strength of the PVA‐cell(1) film was much less than that of the PVA film. It was considered that the PVA‐cell(1) film collapsed at the aggregated part of cells under the external stress due to the fragility of the cell aggregation.

**Table 2:** Young's modulus, tensile strength, and strain at break of PVA‐cell‐plastic films

Film	Young's modulus [GPa]	Tensile strength [MPa]	Strain at break [%]
PVA	1.20 ± 0.05	47.8 ± 7.9	15.5 ± 3.0
PVA‐cell(1)	1.02 ± 0.09	10.1 ± 2.0	1.4 ± 0.4
PVA‐cell(2)	0.57 ± 0.09	3.9 ± 1.2	0.7 ± 0.2
cPVA	2.24 ± 0.27	58.9 ± 21.1	29.8 ± 9.4
cPVA‐cell(1)	1.22 ± 0.22	15.0 ± 2.4	2.0 ± 1.4
cPVA‐cell(2)	0.55 ± 0.13	3.7 ± 1.5	0.9 ± 0.5

In addition, both Young's modulus and the tensile strength of the cPVA and cPVA‐cell(1) films were greater than those of the PVA and PVA‐cell(1) films, respectively. This result indicated that the cross‐linking structure improved the mechanical strength of the film. In contrast, the Young's modulus and tensile strength decreases for PVA‐cell(2) and cPVA‐cell(2) films, suggesting that the mechanical strength of the cell‐plastics decreases as a function of the cell‐to‐matrix weight ratio. In addition, based on stress‐strain curves, the break elongation of PVA and cPVA films ranged from 10% to 40%, whereas the break elongation of each cell‐plastic film was <3%, which suggests that the cell‐plastics are more brittle than commodity plastics. The strain at break values of each PVA‐cell film show quite low compared to those of each PVA film. To improve elongation of the film, it would be necessary to add fillers or plasticizers in the PVA‐cell films. Note that, the mechanical properties of the cell‐plastic films allow their use in daily life because the Young's modulus and tensile strength of PVA‐cell(1) and cPVA‐cell(1) films are competitive with those of commodity plastics.^[^ ^12^ ^]^

### 2.3 Evaluations of Thermal Properties of PVA‐Cell Plastics

Next, we discuss the thermal properties of PVA‐cell‐plastic films based on a thermomechanical analysis (TMA). Figure S4, Supporting Information shows TMA plots for each film loaded at 500 mN; the two fits at relatively low and high temperatures are calculated by using a least squares method. The average coefficient of linear thermal expansion (α_L_ for the low temperature range and α_H_ for the high‐temperature range) are determined based on the slopes of the fits, and the glass transition temperature *T*_g_ is determined from the intersections of the fits. **Table** **3** summarizes these results. The values of *T*_g_ for the non‐crosslinked films remained almost constant. On the other hand, α_L_ for the PVA‐cell(1) and PVA‐cell(2) films slightly exceeded that for the PVA film, whereas α_H_ for the cell‐containing films was much smaller than that for the PVA film. We concluded that the PVA‐cell(1) and PVA‐cell(2) films expanded more easily than the PVA film at low temperature because the cells and the PVA polymer chains interacted less than the polymer chains in PVA. In contrast, at higher temperature, the cell exhibited quite small thermal expansion as comparison to PVA although the thermal mobility of the PVA polymer chains increases at temperatures exceeding *T*_g_.

**Table 3:** Glass transition temperature *T*_g_ and average coefficient of linear thermal expansion below *T*_g_ (α_L_) and above *T*_g_ (α_H_)

Film	α_L_ [×10^−5^ °C^−1^]	*T*_g_ [°C]	α_H_ [×10^−5^ °C^−1^]
PVA	6.35	55	85.9
PVA‐cell(1)	7.39	54	16.8
PVA‐cell(2)	7.54	53	18.0
cPVA	6.54	71	58.8
cPVA‐cell(1)	6.90	57	14.3
cPVA‐cell(2)	8.90	57	21.3

For the cross‐linking PVA films, the values of *T*_g_ were detected at relatively higher temperature than the corresponding non‐crosslinked films, which indicated that the cross‐linking structure increases *T*_g_ due to restricting the thermal mobility of components. Moreover, the *T*_g_ of cPVA was higher than those of cPVA‐cell(1) and cPVA‐cell(2). We assume that the reduction of *T*_g_ of cell‐containing cPVA compared to cPVA is probably because of defect structures of the cPVA matrix due to the addition of the cell or of weak interfacial adhesion between PVA and cell. In contrast, the difference of α_L_ and α_H_ between the cross‐linked films without and with the cell was similar to those of the series of the non‐crosslinking films. Thus, it was found that the addition of the cell and the introduction of the cross‐linking structure to the films affected the thermal expansion of the film.

### 2.4 Investigation of Water‐Absorption Ability of PVA‐Cell Plastics

Next, to examine the possible practical uses of the films, we investigated the water solubility of PVA‐cell‐plastic films. Upon immersing PVA‐cell(1) and PVA‐cell(2) films in water at room temperature, the cell was separated from the film, whereas the cPVA‐cell(1) film maintained its structure in water (Figure S5, Supporting Information). This indicates that the cross‐linking structure is what allows the material to retain the cell‐plastic. Unfortunately, upon immersing cPVA‐cell(2) film in water, the separation of the cell from the film was observed, which was probably due to a lack of cPVA. In addition, water‐immersed PVA‐cell(1) film dissolved within 30 s on a hot plate set at 100 °C, whereas cPVA‐cell(1) did not dissolve in hot water for several hours (Figure S5, Supporting Information). This is again attributed to a decreased solubility due to the cross‐linking structure. The non‐crosslinked films could thus expect to see use as a water‐soluble plastic material like PVA. In addition, applications for the cross‐linked film could exploit its porous structure, which allows it to absorb water like a sponge.

**Figure 6:**
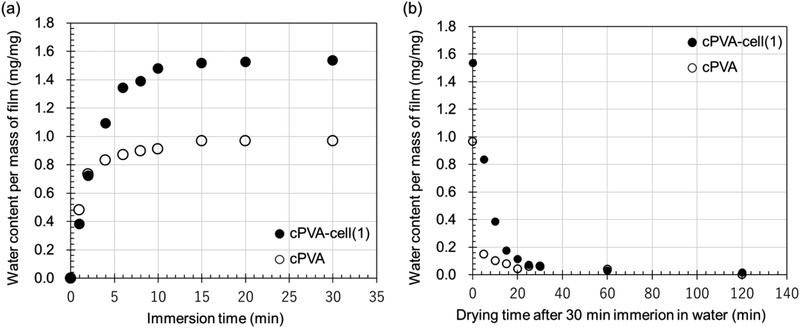
Water content per unit mass of films of cPVA (open circles) and cPVA‐cell(1) (solid circles) as a function of a) water immersion time and b) drying time after water immersion.

**Figure 7:**
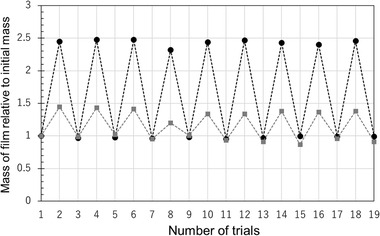
Mass of cPVA‐cell(1) film (black circle) and cPVA film (gray square) relative to their initial mass. The odd and even trial numbers correspond to immersion in water for 30 min and drying at 50 °C for 30 min, respectively.

To evaluate the water absorption capacity of the films, we investigated the water content per unit mass of the cross‐linked film and the drying time of a wet film of the cPVA‐cell(1) film with cPVA as a reference. **Figure** **6**a plots the water content per initial mass of the film as a function of immersion time in water. The water contents of both films reached saturation after roughly 15 min of immersion. Surprisingly, the average water content ratio after 30 min immersion of the cPVA‐cell(1) film was 1.6 ± 0.1, which was almost twice that of the cPVA film. Figure 6b plots the water content of the wet film as a function of drying time at 50 °C in an oven. Within 5 min, the wet cPVA‐cell(1) film loses 50% of water, whereas, for the cPVA film, 80% of water was evaporated in the same time period, suggesting that the former offers better water‐retention. After drying at 50 °C for 30 min, both films lose over 95% of water. The water absorption (30 min immersion) and drying (50 °C for 30 min) measurements were repeated at least nine times for the cPVA‐cell(1) film to investigate their durability under repeated use. As the result, the mass of water absorbed and dried film mass were maintained in all trials (**Figure** **7**), which indicated that the addition of the cell to the film improves the water‐retention ability and the film durability, making it suitable for long‐term use.

## 3 Conclusions

With the goal of developing a carbon‐cycling system for the production and disposal of plastic materials, we propose herein the fabrication of cell‐plastics in which unicellular algal cells serve as primary resources. We manufactured the PVA‐cell‐plastic films by mixing *Chlorella* sp. and PVA having biodegradability and water‐solubility. In addition, we investigated how the mechanical and thermal properties of the film were affected by the cross‐linking of the matrix and the mixing ratio of the cell and PVA. The results indicated that the cross‐linking structure gave high mechanical strength of the PVA‐cell‐plastic films, with high cell content giving low mechanical strength. Moreover, unlike the cell‐plastic films made using PBS as the matrix,^[^ ^7b^ ^]^ the PVA‐cell‐plastic films had mechanical strength competitive with that of the PVA film. Amazingly, the cPVA‐cell(1) film repeated water absorption and drying with the higher water content compared to the cPVA film, which indicated that PVA‐cell‐plastic material was suitable as a functional material. In addition, because the elongation of PVA‐cell films was quite low compared to that of PVA film, the addition of fillers or plasticizers should be considered in the future work although it would be difficult to improve elongation of the films due to the difference of size between cell and polymer. Moreover, it is also necessary to find a way to use the cell‐plastics as materials with a low elongation rate such as .plate materials. These results provide insight into how cell‐matrix affinity, the chemical structures of matrices, and the mixing ratio of components affect the production of functional cell‐plastic materials and bring us closer to environmentally friendly plastics to support a sustainable society in the near future.

## 4 Experimental Section

### Reagents

Commercially available PVA and powdery cell, that is, *Chlorella* sp., were used without further purification. The degree of polymerization and hydrolysis of PVA are ≈1700 and more than 97 mol% of a hydroxy group, respectively.^[^ ^13^ ^]^

### Preparation of PVA‐Cell‐Plastic Films

PVA was dissolved into ultrapure water to obtain 0.1 mg mL^−1^ of a PVA aq. solution. Solutions of 0.5 wt% glutaraldehyde aq. and 6 m H_2_SO_4_ aq. were prepared by dilution of 50 wt% glutaraldehyde aq. and concentrated sulfuric acid, respectively, with ultrapure water. PVA film was prepared by the solution coating method. After coating PVA *aq*. onto a polyimide film (Upilex, Ube Industries, LTD.), a pre‐dried film was obtained after setting at room temperature for three days. PVA film was obtained after further drying in an oven at 100 °C for 4 h.^[^ ^11d^ ^]^ To prepare cPVA film, 500 µL of 0.5 wt% glutaraldehyde aq. (28 mmol) and 100 µL of 6 m H_2_SO_4_ aq. (0.60 mmol) were added to 3 mL of the PVA aq. (PVA: 300 mg). After stirring at room temperature, cPVA film was obtained as done for the PVA film. For PVA‐cell‐plastic and cPVA‐cell‐plastic films, powdery *Chlorella* sp. (300 or 600 mg) was added to 3 mL of the PVA aq. For cPVA‐cell‐plastic film, 500 µL of 0.5 wt% glutaraldehyde aq. (28 mmol) and 100 µL of 6 m H_2_SO_4_ aq. (0.60 mmol) was also added. After stirring at room temperature, PVA‐cell‐plastic and cPVA‐cell‐plastic films were prepared following the same method as above. In addition, tensile test was performed immediately using the sample dried by above method to suppress the influence of humidity.^[^ ^14^ ^]^

## Conflict of Interest

The authors declare no conflict of interest.

## Supporting information

Supporting Information

## Data Availability

Research data are not shared.
