# Supplementary material for: Fabrication of Novel Functional Cell‐Plastic Using Polyvinyl Alcohol: Effects of Cross‐Linking Structure and Mixing Ratio of Components on the Mechanical and Thermal Properties
Source: Glob Chall. 2021 Jun 27;5(8):2100026. doi: 10.1002/gch2.202100026 (PMC8335826; doi:10.1002/gch2.202100026)
Supplement: Supplementary file 1 — Supporting Information [file GCH2-5-2100026-s001.pdf]

© 2021 Wiley-VCH GmbH

Global Challenges Open Access

Supporting Information

for *Global Challenges,* DOI: 10.1002/gch2.202100026

Fabrication of Novel Functional Cell-Plastic Using Polyvinyl Alcohol: Effects of Cross-Linking Structure and Mixing Ratio of Components on the Mechanical and Thermal Properties

Kohei Iritani, Akihito Nakanishi, Ayami Ota, and Takashi Yamashita*

Supporting Information

Fabrication of Novel Functional Cell-Plastic Using Polyvinyl Alcohol: Effects of Cross-Linking Structure and Mixing Ratio of Components on the Mechanical and Thermal Properties

Kohei Iritani[*,†], *Akihito Nakanishi*[†], *Ayami Ota, Takashi Yamashita*

(†Co-first author, *corresponding author)

Dr. K. Iritani, A. Ota, Prof. T. Yamashita

Department of Applied Chemistry

School of Engineering

Tokyo University of Technology

Tokyo 192-0982, Japan

E-mail: iritanikh@stf.teu.ac.jp

Dr. A. Nakanishi

School of Bioscience and Biotechnology,

Tokyo University of Technology,

Tokyo 192-0982, Japan;

Graduate School of Bionics,

Tokyo University of Technology,

Tokyo 192-0982, Japan

1. Experimental Details

1.1. Reagents

All commercially available reagents were procured from Tokyo Chemical Industry Co., Ltd. and Wako Pure Chemical Industries, Ltd., and used without further purification. The degree of polymerization and hydrolysis of PVA are approximately 1,700 and more than 97 mol% of a hydroxy group, respectively. Powdery cells, i.e., *Chlorella* sp., were obtained from Yasashisa Kyoto-kan and used without any chemical or physical treatment. Ultrapure water (18.2 MΩ cm) was produced using ultrapure water production equipment (RFU424BB, ADVANTEC Co. Ltd.) for use as a solvent.

1.2. Optical microscopy imaging of *Chlorella* sp.

Optical microscopy imaging was done by using a CX32PF microscope (Olympus).

1.3. Scanning electron microscopy (SEM) imaging of PVA-cell-plastic films

Before SEM imaging, each PVA or PVA-cell-plastic film was ion coated by using an IB-2 ion coater (Eiko Corporation). SEM images were acquired by using an JSM-6060LV SEM (Japan Electron Optics Laboratory Co., Ltd.).

1.4. Tensile test of films

We used an EX-SX tensile strength tester (Shimadzu) to determine the Young's modulus and tensile strength of the PVA and PVA-cell-plastic films. To fit into the tensile strength tester, the films were cut to 5 mm × 50 mm using a cutter. The crosshead

rate was set to 0.5 mm min^{-1}. The Young's modulus E of the film was calculated by using

$$E = (W/A)/(X/L),$$

where W, A, X, and L are the sample mass, cross section, displacement during tensile test, and length, respectively. The values of W and X were detected by tensile strength tests. The cross section A was calculated as the product of the width (5 mm) and the thickness measured by a film thickness meter. The length L was set to 10 mm for both of samples. In addition, stress and strain are given by W/A and X/L, respectively. The stress was plotted as a function of strain for the tensile strength tests.

1.5. Contact angle

A DropMaster 300 (Kyowa Interface Science Co., Ltd.) served to evaluate the contact angle, which was determined by depositing a drop of water on the film. All pictures were taken within 1 min after dropping water on the film surface.

1.6. Water absorption and desorption of PVA-cell-plastic film

After measuring the mass of a dried film, it was immersed in ultrapure water for a given period of time. The excessive water attached to the surface was removed by

swiping with Kimwipes, following which the mass of the wet film was measured. The water content W_w of the film per unit mass of film was calculated by using

$$W_w = (W_f - W_s)/W_s,$$

where W_s and W_f are the initial mass of the dry film and the mass of the wet film, respectively.

1.7. Thermomechanical analysis of films

TMA was carried out by using a Thermo plus EVO2 TMA8311 (Rigaku). To fit the tester, the films were cut to 5 mm × 25 mm by using a cutter. The average coefficient α of linear thermal expansion of the film was calculated by using

$$\alpha = k/L,$$

where k and is the slope of the line fit to the TMA curve by using the least squares method and L is the initial length of the film, as measured by the tester. Because two linear fits were obtained with differing slopes, two coefficients α_L and α_H were calculated. The glass transition temperature T_g delimits the two fitting regions.

2. Additional Images

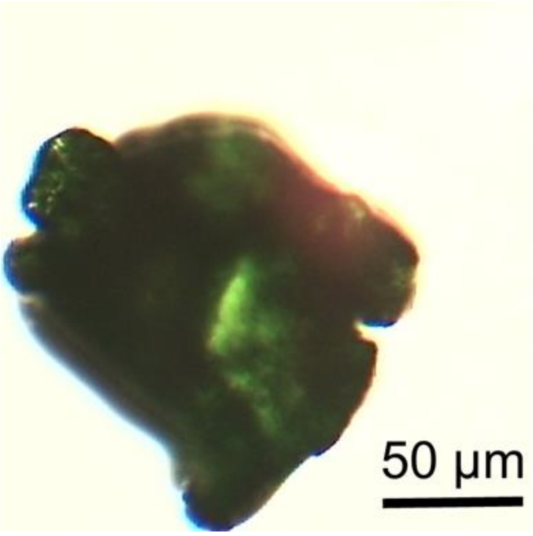

Figure S1. Typical optical microscopy image of chlorella with large size.

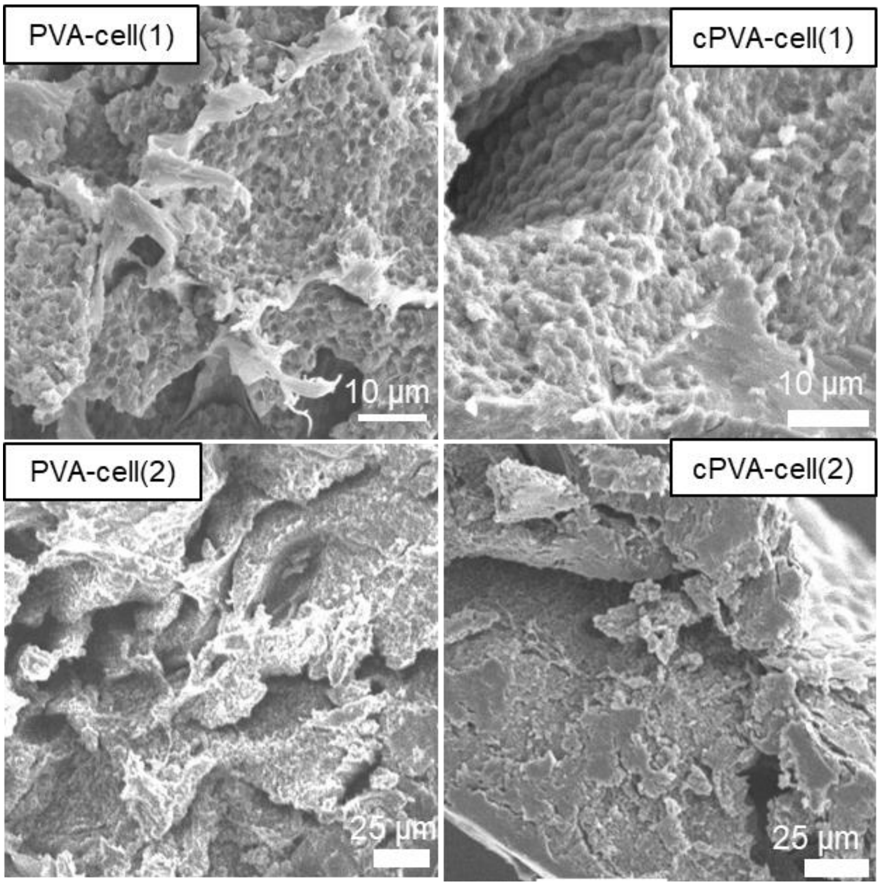

Figure S2. SEM images of cross sections of each PVA-cell plastic films.

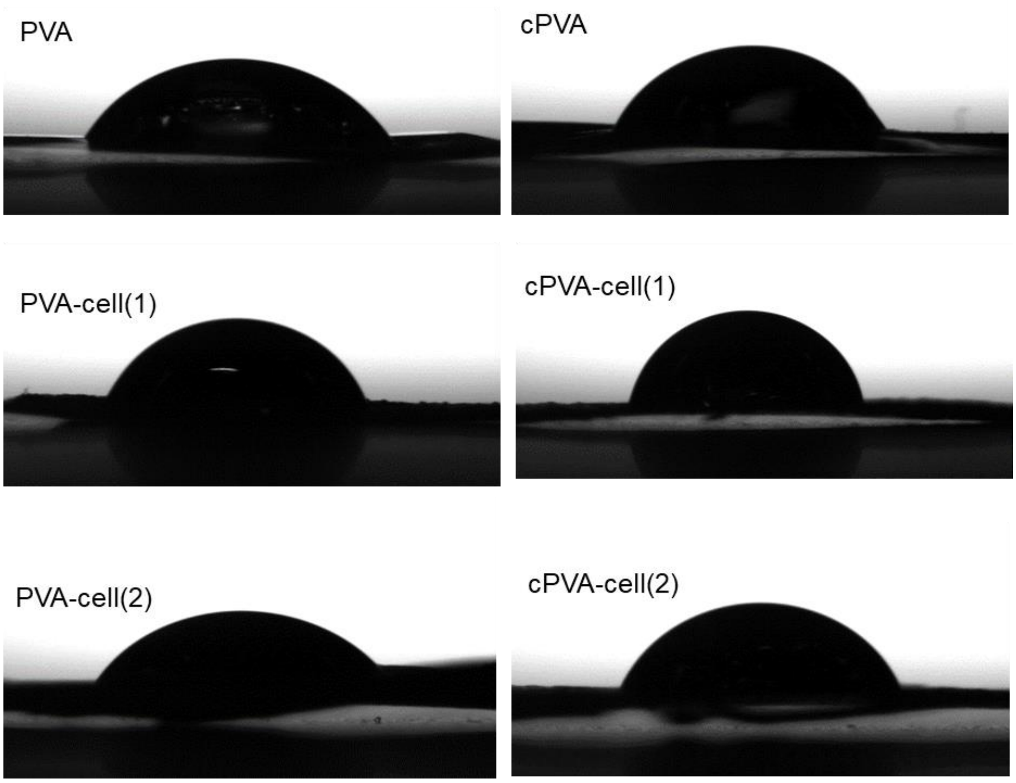

Figure S3. Photo images of each PVA-cell plastic film after dropwise of a drop of water.

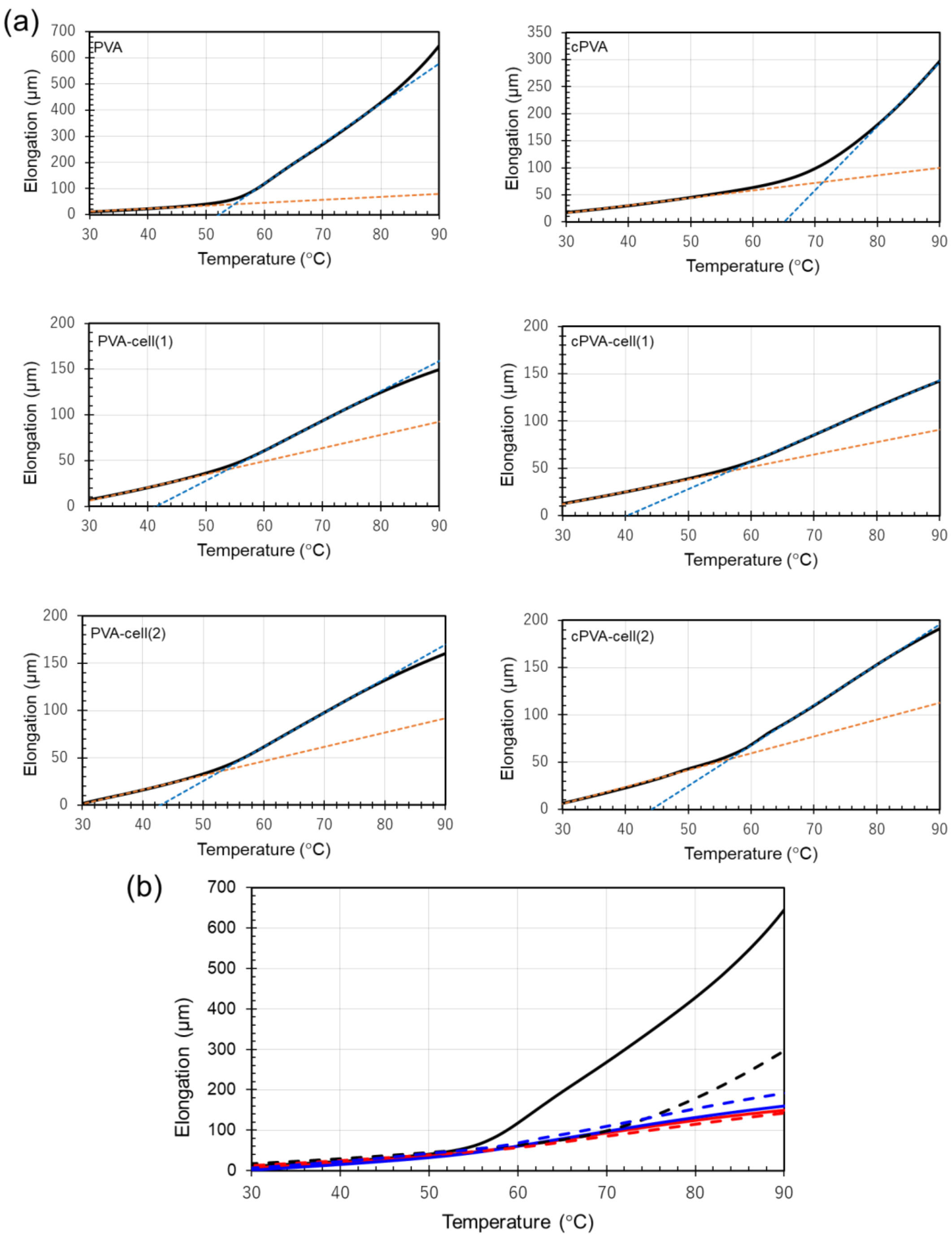

Figure S4. (a) TMA curves of each film (black lines). The orange and blue dotted lines shows fitting lines calculated by least squares method. (b) Superimposed data of all the curves of S4a: black line; PVA, red line; PVA-cell(1), blue line; PVA-cell(2), black dashed line; cPVA, red dashed line; cPVA-cell(1), blue dashed line; cPVA-cell(2).

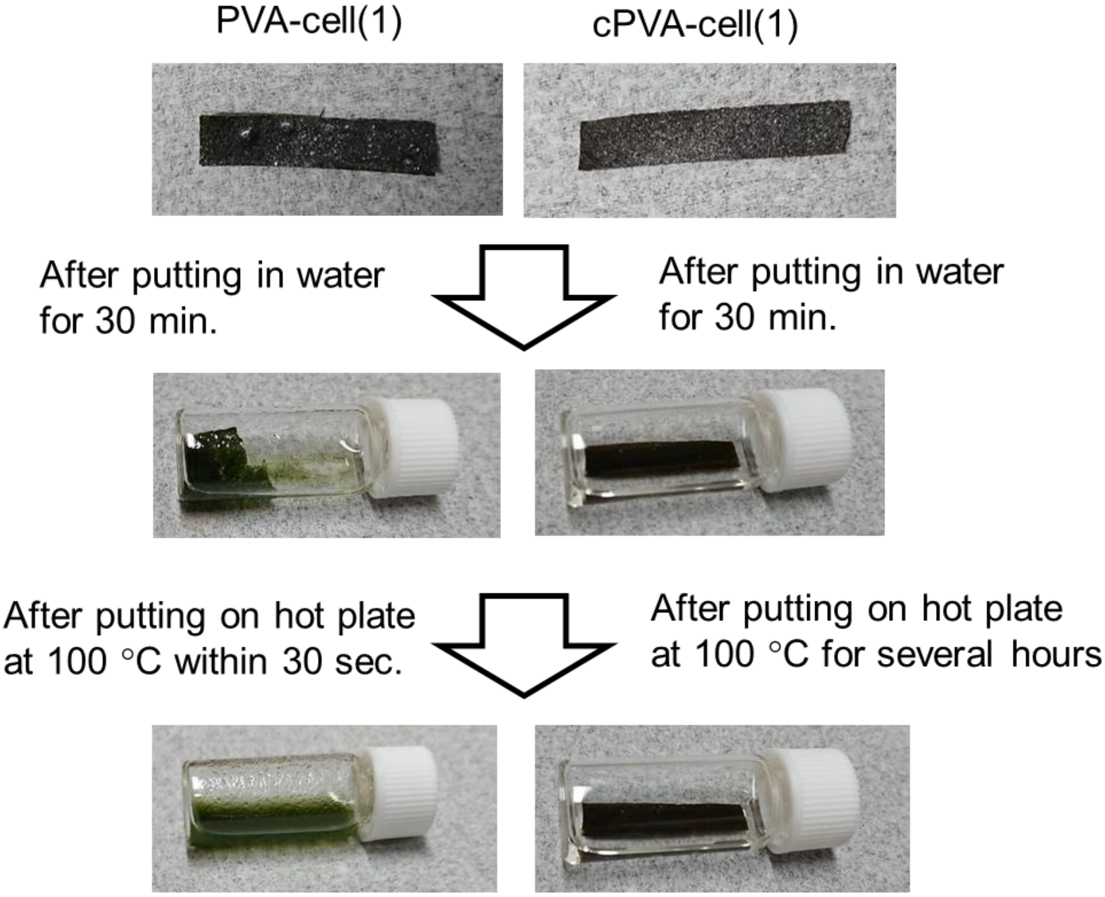

Figure S5. Photo images of (top) PVA-cell(1) and cPVA-cell(1), (middle) those after putting in water for 30 min, (bottom) those after putting on a hot plate at 100°C within 30 sec (for PVA-cell(1)) and for several hours (cPVA-cell(1)).
